# miR-137 prevents inflammatory response, oxidative stress, neuronal injury and cognitive impairment via blockade of *Src*-mediated MAPK signaling pathway in ischemic stroke

**DOI:** 10.18632/aging.103301

**Published:** 2020-06-04

**Authors:** Runhui Tian, Bo Wu, Cong Fu, Kaimin Guo

**Affiliations:** 1 Department of Psychology, The First Hospital of Jilin University, Changchun 130021, P.R. China; 2 Department of Psychology, The Sixth People's Hospital of Changchun, Changchun 130000, P.R. China; 3 Department of Andrology, The First Hospital of Jilin University, Changchun 130021, P.R. China

**Keywords:** ischemic stroke, astrocytes, neuron, microRNA-137, gene *Src*

## Abstract

Stroke is a leading cause of death and disability worldwide. The purpose of this study was to investigate the possible role of the microRNA (miRNA or miR) miR-137 in ischemic stroke. miRNAs are very stable in the blood and may serve as potential diagnostic and therapeutic markers. Wild-type, *Src*^-/-^ and miR-137^-/-^ mice were treated with *p38* siRNA or *Erk2* siRNA to identify their roles in the inflammatory response, oxidative stress, neuronal injury and cognitive impairment in brain tissues of mice following middle cerebral artery occlusion (MCAO) operation. We evaluated several factors including; inflammatory responses, oxidative stress, viability and apoptosis of astrocytes in order to identify the functions of miR-137 and *Src* in ischemic stroke. miR-137 alleviated the inflammatory response, oxidative stress, neuronal injury and cognitive impairment, and restricted apoptosis via targeting *Src* and inactivating the MAPK signaling pathway. Furthermore, up-regulation of miR-137 or inhibition of *Src* inhibited the secretion of inflammatory factors, suppressed oxidative stress, and reduced apoptosis of astrocytes. In conclusion, our work suggests that, in mice, miR-137 confers neuroprotective effects against ischemic stroke via attenuation of oxidative, apoptotic, and inflammatory pathways through inhibiting *Src*-dependent MAPK signaling pathway.

## INTRODUCTION

Stroke is one of the leading causes of death and long-term disability in adults [1]. The brain consumes more than 20% of the total energy produced by the body and stroke alters the energy balance in the brain, causing brain damage, such as neuronal injury, neuroinflammation, and cerebral edema [2]. Over 80% of stroke cases are ischemic stroke, which results from a loss of blood supply to the brain due to the blockage of a blood vessel [3]. Ischemic stroke is a common central nervous system disease but effective treatment strategies are lacking. It causes approximately 4.4 million deaths each year and treating patients who have suffered a stroke generates between $59 and $230 thousand dollars in health care expenses per patient, putting severe economic burdens on patients, families, and national health services [4]. Ischemic stroke is a multi-factorial disease associated with inflammation, apoptosis, and oxidative stress responses [5]. The subsequent oxidative stress that follows ischemic stroke causes neuronal death, damage to the blood-brain barrier, neurovascular injury, edema and bleeding [6].

MicroRNAs (miRNAs) regulate cellular pathways in a variety of clinical disorders, including ischemic stroke [7]. miR-137 is enriched in the brain and negatively regulates cell proliferation and accelerates the neuronal differentiation of embryonic neural stem cells [8]. Furthermore, miR-137 promotes endothelial progenitor cell proliferation and angiogenesis in mice with cerebral ischemic stroke by targeting NR4A2 [9]. In addition, miR-137 confers protection against ischemia/reperfusion injury in neurons [10]. Notably, miR-137 has complementarity to the 3’-untranslated region (3’UTR) of *Src* mRNA and can suppress *Src*-related oncogenic signaling, thus decelerating cancer progression [11]. *Src* is stimulated by many cytokines and growth factors like TGF-β1 and EGF, resulting in autophosphorylation of Tyr416 [12]. Silencing the *Src* gene as well as inhibiting *Src*-family kinases (SFKs) significantly decreases brain injury after cerebral- or hypoxia-induced ischemia [13–15]. Moreover, *Src* regulates the activation of the cecum-specific mitogen-activated protein kinase (MAPK) signaling pathway and inflammation during cecal tumorigenesis [16]. MAPK is a highly conserved pathway controlled by a cascade of protein kinases and MAPK phosphatases that regulates the cellular response to various extracellular stimuli, ranging from growth factors to environmental stresses [17]. Current findings have also demonstrated that activated MAPK signaling pathway contributes to the activation of the NLRP1 and NLRP3 inflammasome proteins in neurons and brain tissues following ischemic stroke [18]. Based on the aforementioned findings, we hypothesized that miR-137 may play a significant role in regulating the response ischemic stroke by manipulating the *Src* and MAPK signaling pathway. Therefore, we conducted several experiments to investigate the mechanism by which the miR-137/*Src/*MAPK signaling pathway affects ischemic stroke.

## RESULTS

### Bioinformatics analysis to predict the differentially expressed genes (DEGs) and their molecular interactions in ischemic stroke

**Figure 1:**
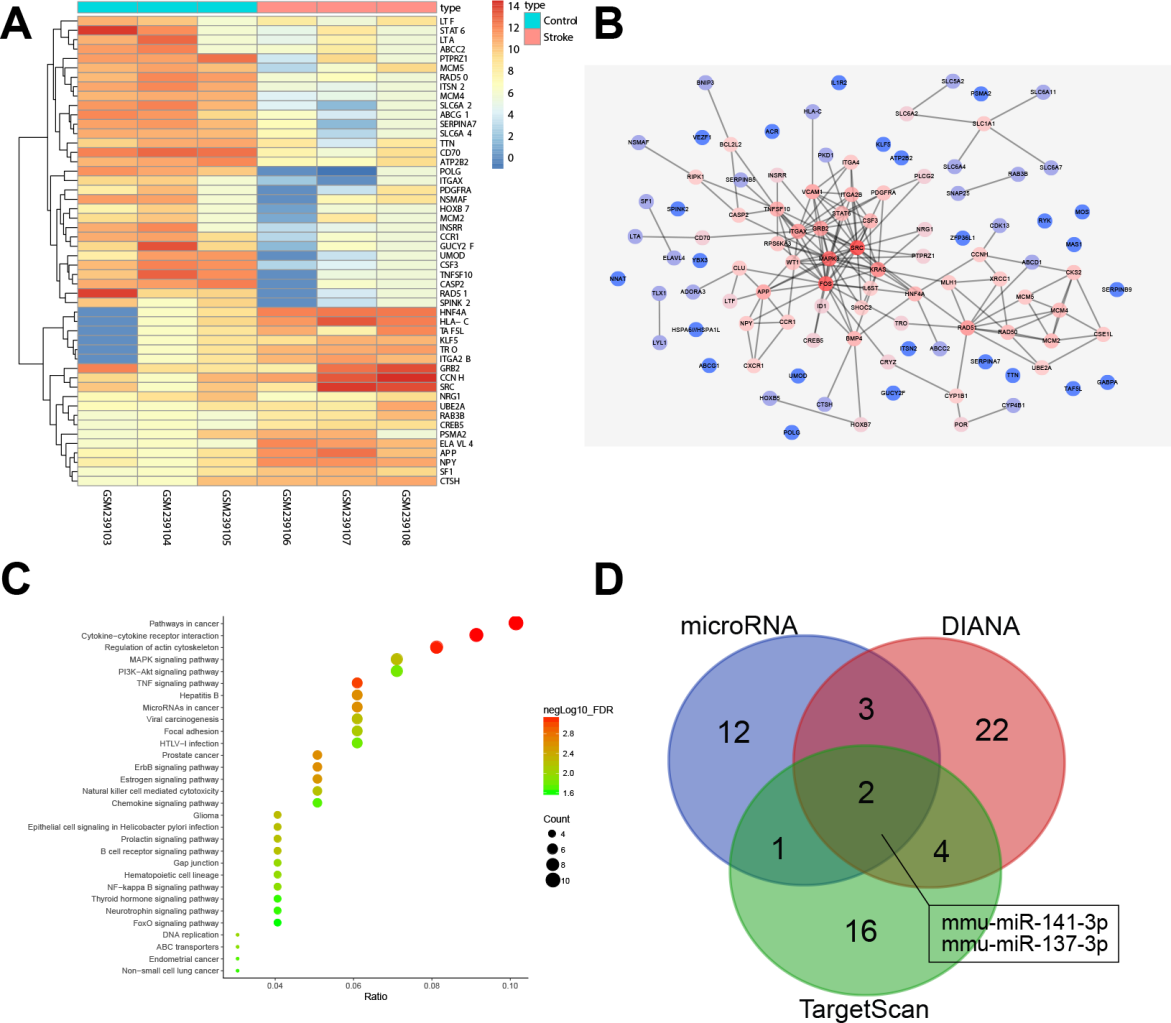
**miRNA and mRNA expression profiles in ischemic stroke.** (**A**) a heat map of the expression of the first 50 DEGs in ischemic stroke-related microarray data GSE9391. The colored column represents the sample number, the row name indicates the DEGs, each rectangle in the graph corresponds to the expression value of a sample, red indicates high expression and blue indicates poor express; (**B**) PPI network for the DEGs in ischemic stroke. The color of genes indicates the degree of association with other genes and, if a gene has more interacting genes, the core degree of the gene in the network and the correlation degree will be higher; red indicates high association and blue indicates low association; (**C**) enrichment analysis of KEGG for DEGs in ischemic stroke; the abscissa represents gene ratio and the coordinate represents KEGG enrichment item; the right histogram represents color gradation; (**D**) comparisons among miRNAs targeting *Src* by DIANA, TargetScan and microRNA. Two miRNAs were located at the intersection of the three databases (mmu-miR-141-3p and mmu-miR-137-3p).

Figure 1A shows a heat map displaying the expression of the first 50 DEGs identified in the ischemic stroke-associated microarray data, GSE9391. In addition, a protein-protein interaction (PPI) network containing the first 100 DEGs was generated (Figure 1B), which identified *Src* as the gene having the highest degree of association with other genes and suggesting that *Src* may affect ischemic stroke. Analysis of the Kyoto Encyclopedia of Genes and Genomes (KEGG), from the KEGG database (http://www.genome.jp/kegg/pathway.html), for DEGs (Figure 1C) and the PPI network diagram (Figure 1B) showed that *Src* associated with PDGFRA, RPS6KA3/MAPK, GRB2 and especially, the MAPK signaling pathway, indicating that *Src* may affect the MAPK signaling pathway. The *Src-*related signaling pathways were identified in the KEGG analysis, which showed that *Src* was located upstream of the MAPK signaling pathway in GnRH signaling pathway (map04912). Thus, we speculated that *Src* may regulate the MAPK signaling pathway in ischemic stroke. miRNAs that could potentially target *Src* were predicted and analyzed by the DIANA, TargetScan and microRNA databases. A total of 31, 23 and 18 miRNAs were obtained from each database, respectively. A Venn diagram was drawn to identify genes at the intersection of the 3 miRNA databases (Figure 1D). Only two miRNAs, mmu-miR-141-3p and mmu-miR-137-3p, were identified by the three databases, indicating that these two miRNAs were very likely to regulate *Src*. Based on the above analysis, we chose to investigate the role of miR-137 in regulating the *Src*-mediated MAPK signaling pathway during ischemic stroke.

### Inhibition of *p38* or *Erk2* alleviates cerebral infarction, improves neuronal functions, and enhances learning and memory abilities in middle cerebral artery occlusion (MCAO) mice

Next, we sought to test whether the MAPK signaling pathway affects the progression of ischemic stroke in mice. At first, the efficiency of *p38* or *Erk2* knockdown was verified in isolated astrocytes. The astrocytes were stained for glial fibrillary acidic protein (GFAP) expression and observed under an inverted microscope. The tetramethyl rhodamine isothiocyanate (TRITC)-positive cells were red, namely astrocytes and the astrocytes accounted for more than 95% of the total cells (Supplementary Figure 1A, 1B). Next, the results of reverse transcription quantitative polymerase chain reaction (RT-qPCR) showed significantly reduced expression of *p38* in cells upon treatment with si-*p38*-1, si-*p38*-2 and si-*p38*-3, with si-*p38*-1 exhibiting the lowest *p38* expression. Therefore, si-*p38*-1 was selected for subsequent experiments (Supplementary Figure 1C). Similarly, the expression of *Erk2* was decreased in cells treated with si-*Erk2*-1, si-*Erk2*-2 and si-*Erk2*-3, with si-*Erk2*-1 exhibiting the lowest *Erk2* expression, so si-*Erk2*-1 was selected for subsequent experiments (Supplementary Figure 1D).

**Figure 2:**
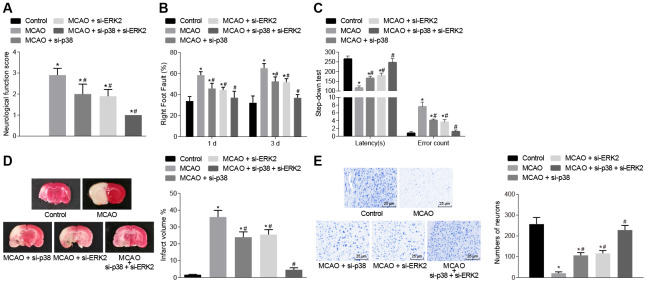
**Inhibition of *p38* or *Erk2* ameliorates cerebral infarction and improves neuronal function, learning, and memory abilities in MCAO mice.** (**A**) neurological function scores in mice in each group; (**B**) rate of left limb fault step of mice after treatment with si-*p38*, si-*Erk2* or in combination; (**C**) latency period and number of errors of mice treated with si-*p38*, si-*Erk2* or in combination; (**D**) changes in the volume of infarct in mice treated with si-*p38*, si-*Erk2* or in combination using TTC staining; (**E**) changes in the number of neurons from mice treated with si-*p38*, si-*Erk2* or in combination using Nissl staining (× 400). Measurement data are expressed as mean ± standard deviation and compared by one-way ANOVA, followed by Tukey's post hoc test; * *p* < 0.05 *vs*. control group (sham-operated wild-type mice); # *p* < 0.05 *vs*. MCAO group (wild-type mouse models of MCAO). N = 15.

The mice then underwent a series of behavioral tests including, nerve function scores, the foot-fault test and the step-down test (Figure 2A–2C). Wild-type mouse models of MCAO treated with lentivirus exhibited increased neurological function scores, number of errors, and rate of left limb fault step, and shorter latency period compared with sham-operated mice (control mice), (*p* < 0.05). This observation was eliminated in the mice treated with *p38* small interfering RNA (siRNA) or *Erk2* siRNA.

The volume of infarct in wild-type mouse models of MCAO treated with lentivirus was measured after staining with 2, 3, 5-triphenyltetrazolium chloride (TTC) or Nissl (Figure 2D–2E). The volume of infarct was increased (*p* < 0.05) compared to control mice but the number of neurons showed no significant difference (*p* > 0.05). Wild-type mice treated with *p38* siRNA or *Erk2* siRNA showed the opposite outcomes (*p* < 0.05). These results demonstrated that inhibition of the p38 or ERK2 signaling pathway alleviated cerebral infarction and neuron damage, cognitive impairment, and improved learning and memory abilities, as well as reduced the score of neurological function injury in MCAO mice.

### Inhibition of *p38* or *Erk2* reduces oxidative stress and inflammatory response in the brain tissues of MCAO mice

**Figure 3:**
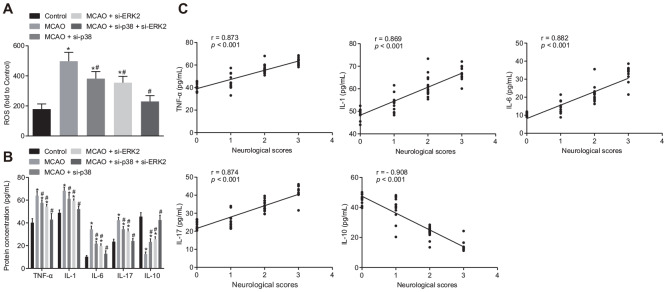
**Inhibition of p38 or ERK2 signaling pathway attenuates oxidative stress and the inflammatory response in the brain of MCAO mice.** (**A**) detection of ROS in brain tissues from mice treated with si-*p38*, si-*Erk2* or in combination using DCFH-DA fluorescent probe; (**B**) detection of TNF-α, IL-1, IL-6, IL-17 and IL-10 protein expression in the brain tissues of mice treated with si-*p38*, si-*Erk2* or in combination by ELISA; (**C**) correlation analysis between the score of neurological function and contents of TNF-α, IL-1, IL-6, IL-17 and IL-10 in brain tissues of mice treated with si-*p38*, si-*Erk2* or in combination. Measurement data are expressed as mean ± standard deviation, which were compared by one-way ANOVA, followed by Tukey's post hoc test. Correlation analysis was performed using Pearson correlation coefficient, r > 0 represents a positive correlation, r < 0 represents a negative correlation, *p* < 0.05 was indicative of statistical significance; * *p* < 0.05 *vs*. control group (sham-operated wild-type mice); # *p* < 0.05 *vs*. MCAO group (wild-type mouse models of MCAO). N = 15.

Next, we measured the levels of reactive oxygen species (ROS) and inflammatory factors in mouse brain tissues. The levels of ROS, tumor necrosis factor-α (TNF-α), interleukin (IL)-1, IL-6 and IL-17 were increased in comparison to control mice (Figure 3A, 3B), while IL-10 concentration was decreased in brain tissues of wild-type mouse models of MCAO treated with lentivirus (*p* < 0.05). Treatment with si-*p38* or si-*Erk2* reversed the effects of lentiviral treatment (*p* < 0.05). Correlation analysis (Figure 3C) showed that neurological function score was positively correlated with the expression of TNF-α, IL-1, IL-6 and IL-17 (r > 0, *p* < 0.05), and negatively correlated with IL-10 expression (r < 0, *p* < 0.05). Inhibition of the p38 or ERK2 signaling pathway reduced oxidative stress and inflammation in MCAO mice, thereby improving neuronal injury and learning and memory abilities in mice.

### Inhibition of *p38* or *Erk2* attenuates cell apoptosis in brain tissues of MCAO mice

**Figure 4:**
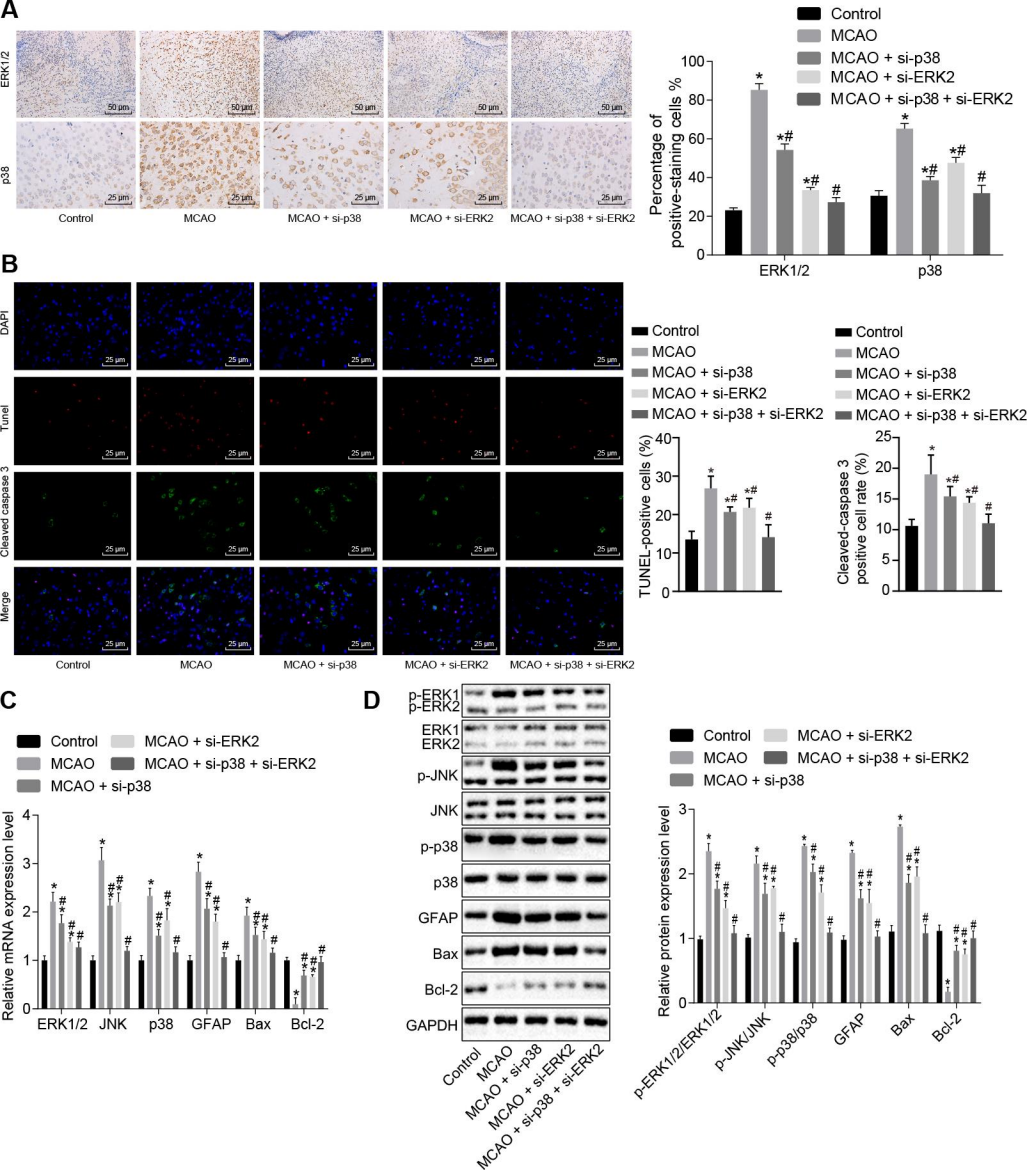
**Inhibition of the p38 or ERK2 signaling pathway decreases cell apoptosis in brain tissues of MCAO mice.** (**A**) ERK1/2 and p38 expression levels in the brain tissues of mice after treatment with si-*p38*, si-*Erk2* alone or in combination were determined by immunohistochemistry (× 200); (**B**) immunofluorescence of cleaved caspase 3, TUNEL staining, and DAPI staining in brain tissues from mice treated with si-*p38* or si-*Erk2* alone or in combination (× 400); (**C**) mRNA levels of *Erk2*, *Jnk*, *p38*, *Gfap*, *Bax* or *Bcl-2* in brain tissues of mice treated with si-*p38* or si-*Erk2* alone or in combination detected using RT-qPCR; (**D**) protein level of GFAP, Bax and Bcl-2 along with the extent of ERK1/2, JNK and p38 phosphorylation in brain tissues of mice treated with si-*p38* or si-*Erk2* alone or in combination detected using Western blot analysis. Measurement data are expressed as mean ± standard deviation and compared using one-way ANOVA, followed by Tukey's post hoc test. * *p* < 0.05 *vs.* control group (sham-operated wild-type mice); # *p* < 0.05 *vs.* MCAO group (wild-type mouse models of MCAO). N = 15.

Next, we sought to examine the effect of the p38 or ERK2 signaling pathway on cell apoptosis. Immunohistochemistry showed that (Figure 4A) ERK1/2 was mainly located in the cell nucleus, and p38 was mainly located in the cytoplasm. Compared with control mice, wild-type mouse models of MCAO treated with lentivirus exhibited a significantly higher number of ERK1/2 and p38 positive cells (*p* < 0.05); however, wild-type mice treated with *p38* siRNA or *Erk2* siRNA exhibited opposite results (*p* < 0.05).

Apoptosis was assessed by staining cells for cleaved caspase 3 (green), TUNEL staining (red) and DAPI staining (blue) (Figure 4B). Cleaved caspase 3 was found primarily in the cytoplasm, whereas TUNEL and DAPI staining were localized to the nucleus. The number of cleaved caspase 3/ TUNEL double positive cells was elevated in wild-type mouse models of MCAO treated with lentivirus, but reduced in wild-type mice treated with *p38* siRNA or *Erk2* siRNA in comparison to control mice (*p* < 0.05).

RT-qPCR and Western blot analysis (Figure 4C, 4D) showed that MCAO-treated wild-type mice exhibited increased expression of ERK1/2, Jun amino-terminal kinase (JNK), p38, GFAP and B-cell lymphoma 2 (Bcl-2)-associated X protein (Bax). Phosphorylation of ERK1/2, JNK and p38 was also increased; however, Bcl-2 was down-regulated in comparison to control mice (*p* < 0.05). Wild-type mice treated with *p38* siRNA or *Erk2* siRNA showed the opposite results when compared with control mice (*p* < 0.05). These results suggest that the MAPK signaling pathway is activated in MCAO-treated mice leading to increased apoptosis. Inhibition of *Erk2* and *p38* diminished levels of *Gfap* and *Bax*, and activated *Bcl-2*, thus inhibiting cell apoptosis.

### Knockout of *Src* gene alleviates cerebral infarction and improves neuron functions as well as learning and memory abilities in MCAO mice

**Figure 5:**
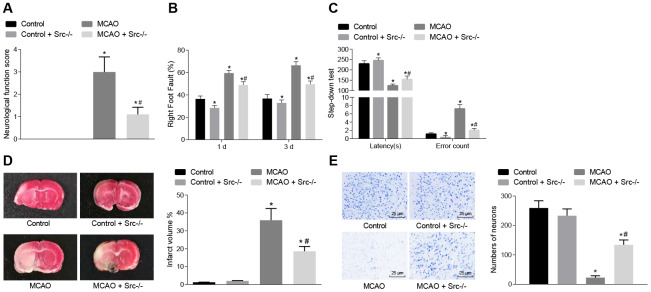
**Knockout of the *Src* gene alleviates cerebral infarction, and improves neuron functions and learning and memory abilities in MCAO mice.** (**A**) neurological function score of *Src*^-/-^ mice. (**B**) rate of left limb fault step in *Src*^-/-^ mice in the foot-fault test; (**C**) latency period and number of errors of *Src*^-/-^ mice during the step-down test; (**D**) the volume of infarct in *Src*^-/-^ mice after TTC staining; (**E**) the number of neurons in *Src*^-/-^ mice using Nissl staining (× 400). Measurement data are expressed as mean ± standard deviation and compared using one-way ANOVA, followed by Tukey's post hoc test. * *p* < 0.05 *vs*. control group (sham-operated wild-type mice); # *p* < 0.05 *vs*. MCAO group (wild-type mouse models of MCAO). N = 15.

In the following experiments, nerve function scoring, foot-fault test results, the step-down test, TTC staining and Nissl staining were used to investigate the role of *Src* in regulating cerebral infarction, neuron function and learning and memory in MCAO mice. Nerve function scoring, foot-fault test and step-down test (Figure 5A–5C) showed that wild-type mouse models of MCAO exhibited higher neurological function scores, number of errors, and rate of left limb fault step, and shorter latency period (*p* < 0.05), while it was reversed in *Src*^-/-^ mice (*p* < 0.05).

TTC staining and Nissl staining were then used for morphological observation, and the results (Figure 5D, 5E) showed that the volume of infarct was increased (*p* < 0.05), but the number of neurons was decreased (*p* < 0.05) in wild-type mouse models of MCAO in comparison to sham-operated wild-type mice. Src knockout (*Src*^-/-^)-treated mice showed the opposite results (*p* < 0.05). The aforementioned results suggest that knockout of the *Src* gene alleviated cerebral infarction and neuron damage, cognitive impairment, and improved learning and memory abilities, and reduced the score of neurological function injury in MCAO mice.

### Knockout of *Src* gene reduces oxidative stress and inflammatory response in the brain tissues of MCAO mice

**Figure 6:**
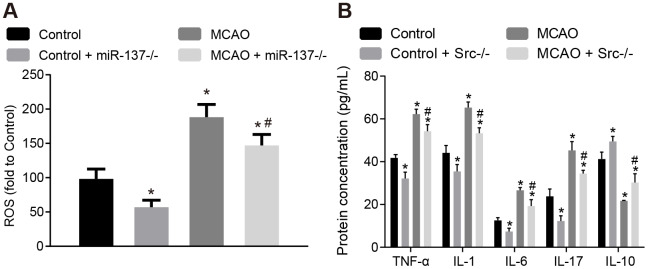
**Knockout of *Src* gene restricts oxidative stress and the expression of inflammatory markers in the brains of MCAO mice.** (**A**) ROS content in the brain tissues of *Src*^-/-^ mice was measured using a DCFH-DA fluorescent probe; (**B**) TNF-α, IL-1, IL-6, IL-17 and IL-10 expression in the brain tissues of *Src*^-/-^ mice using ELISA. Measurement data are expressed as mean ± standard deviation and compared by one-way ANOVA, followed by Tukey's post hoc test. * *p* < 0.05 *vs*. control group (sham-operated wild-type mice); # *p* < 0.05 *vs*. MCAO group (wild-type mouse models of MCAO); N = 15.

ROS and the expression of inflammatory factors were measured in brain tissues (Figure 6). Wild-type mouse models of MCAO displayed increased levels of ROS and increased TNF-α, IL-1, IL-6 and IL-17 expression, whereas IL-10 expression was decreased in brain tissues compared to sham-operated wild-type mice (*p* < 0.05), while *Src*^-/-^ mice showed opposite result (*p* < 0.05). This suggests that knock down *Src* gene expression alleviates oxidative stress and inflammation in MCAO mice, thereby preventing neuronal injury and improving learning and memory in mice.

### Knockout of *Src* gene inactivates the MAPK signaling pathway and reduces cell apoptosis in brain tissues of MCAO mice

**Figure 7:**
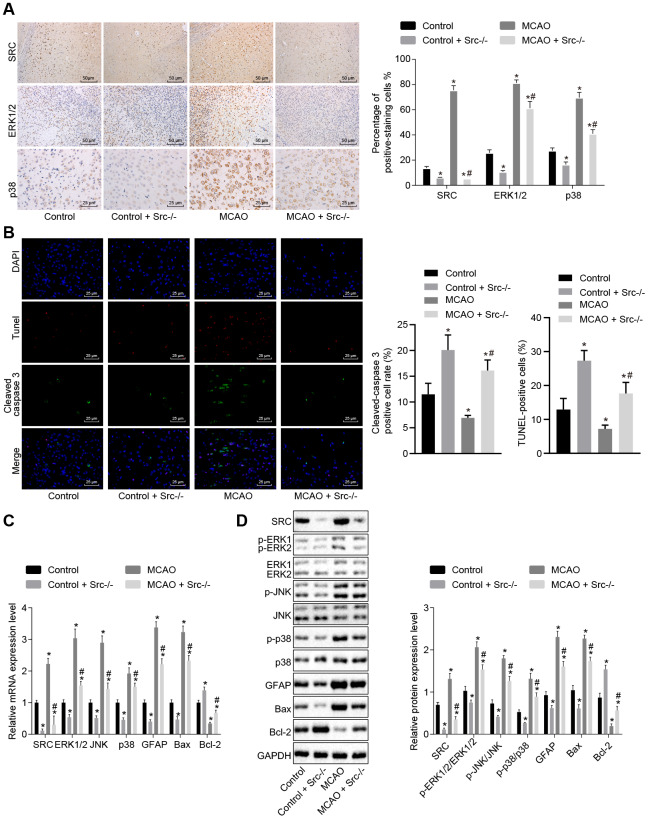
***Src* knockdown reduces cell apoptosis in brain tissues of MCAO mice by down-regulating the MAPK signaling pathway.** (**A**) Immunohistochemistry of ERK1/2 (× 400), p38 (× 400) and Src (× 200) in the brain tissues of *Src*^-/-^ mice; (**B**) diagram based on the immunofluorescence of cleaved caspase 3, TUNEL staining, and DAPI in brain tissues of *Src*^-/-^ mice (× 400); (**C**) mRNA expression of *Erk2*, *Jnk*, *p38*, *Gfap*, *Bax* and *Bcl-2* in brain tissues of *Src*^-/-^ mice using RT-qPCR; (**D**) protein level of GFAP, Bax and Bcl-2 along with the extent of ERK1/2, JNK and p38 phosphorylation in brain tissues of *Src*^-/-^ mice using Western blot analysis. Measurement data are expressed as mean ± standard deviation and compared using one-way ANOVA, followed by Tukey's post hoc test. * *p* < 0.05 *vs.* control group (sham-operated wild-type mice); # *p* < 0.05 *vs.* MCAO group (wild-type mouse models of MCAO). N = 15.

Next, immunohistochemistry was performed and the results showed that Src protein was mainly located in the cell nucleus (Figure 7A). More cells stained positive for ERK1/2, p38 and Src in wild-type mouse models of MCAO compared with sham-operated wild-type mice (*p* < 0.05); however, staining was decreased in *Src*^-/-^ mice (*p* < 0.05). Immunofluorescence analysis (Figure 7B) revealed that the number of cleaved caspase 3^+^ and TUNEL^+^ cells was elevated in wild-type mouse models of MCAO but reduced in *Src*^-/-^ mice in comparison to sham-operated wild-type mice (*p* < 0.05).

RT-qPCR and Western blot analysis (Figure 7C, 7D) showed that wild-type mouse models of MCAO exhibited elevated levels of Src, ERK1/2, JNK, p38, GFAP and Bax as well as increased ERK1/2, JNK and p38 phosphorylation, while the levels of Bcl-2 were diminished. *Src* knockout reversed the effects of MCAO treatment (*p* < 0.05). Based on these results, we concluded that knockout of the *Src* gene inhibited the MAPK signaling pathway and reduced cell apoptosis by suppressing *Gfap* and *Bax* expression while increasing *Bcl-2* expression.

### miR-137 targets *Src* and inhibits its expression

**Figure 8:**
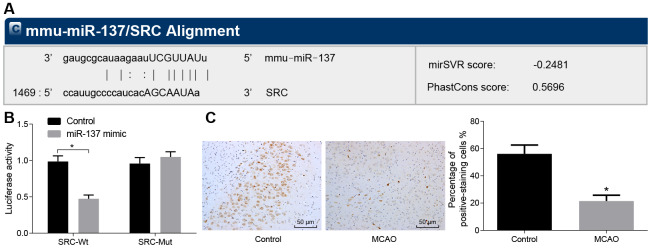
***Src* is a target gene of miR-137.** (**A**) binding site prediction for miR-137: The 3’UTR of *Src* was analyzed using microRNA.org; (**B**) identification of the interaction between miR-137 and *Src* using dual-luciferase reporter assay; (**C**) miR-137 expression in the brain tissues of MCAO mice visualized by *in situ* hybridization (× 200). All measurement data were expressed as mean ± standard deviation and the differences between two groups were compared by independent sample *t*-test. * *p* < 0.05 *vs.* control cells. N = 15.

In the subsequent experiments, we studied the relationship between miR-137 and *Src* gene. Bioinformatic analysis using the website microRNA.org, predicted that miR-137 could target the *Src* gene (Figure 8A). Dual-luciferase reporter assay (Figure 8B) confirmed this interaction as the luciferase activity of *Src* wild-type 3’UTR was significantly inhibited by miR-137 mimic (*p* < 0.05) compared with sham-operated wild-type mice. The luciferase activity of mutant 3’UTR was not impacted by the addition of miR-137 mimic (*p* > 0.05). In situ hybridization (Figure 8C) showed that positive staining for miR-137 probe was purple and granular and was widely distributed in the cerebral cortex and hippocampus. Wild-type mouse models of MCAO exhibited lower miR-137 expression in brain tissues than sham-operated wild-type mice. These findings showed that *Src* was downregulated by miR-13, and that miR-137 was poorly expressed in the brain tissue of MCAO mice.

### Knockout of miR-137 ameliorates cerebral infarction, neuron functions and learning and memory abilities in MCAO mice

**Figure 9:**
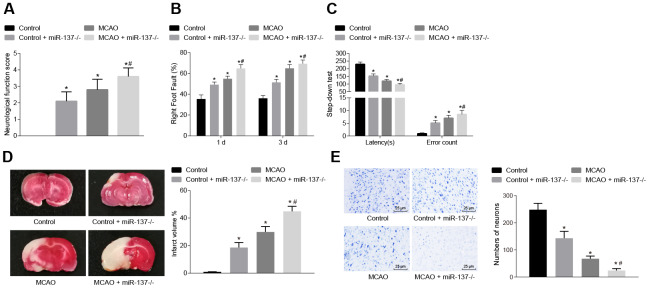
**Downregulated miR-137 accelerates cerebral infarction and impairs neuronal functions and learning and memory abilities in MCAO mice.** (**A**) neurological function scores in miR-137^-/-^ mice; (**B**) rate of left limb fault step of miR-137^-/-^ mice by foot-fault test; (**C**) latency period and number of errors of miR-137^-/-^ mice in the step-down test; (**D**) changes in volume of infarct of miR-137^-/-^ mice using TTC staining; (**E**) changes in number of neurons in miR-137^-/-^ mice using Nissl staining (× 400). Measurement data are expressed as mean ± standard deviation and compared using one-way ANOVA, followed by Tukey's post hoc test. * *p* < 0.05 *vs*. control group (sham-operated wild-type mice); # *p* < 0.05 *vs*. MCAO group (wild-type mouse models of MCAO). N = 15.

We then examined the effects of MCAO treatment and miR-137 on behavioral function. Nerve function scoring, foot-fault test, step-down test, TTC staining and Nissl staining were used to determine the effect of miR-137 on cerebral infarction, neuron functions and learning and memory abilities in MCAO mice. MCAO- and miR-137 knockout (miR-137^-/-^)-treated mice exhibited increased neurological function scores, number of errors, and rate of left limb fault step, yet shorter latency period compared with sham-operated wild-type mice (Figure 9A–9C) (*p* < 0.05).

TTC staining and Nissl staining (Figure 9D, 9E) showed that the volume of infarct was increased (*p* < 0.05), while the number of neurons was decreased (*p* < 0.05) in wild-type mouse models of MCAO in comparison to sham-operated wild-type mice. Both the volume of infarct and number of neurons were reduced in the absence of miR-137 (*p* < 0.05). These results demonstrated that knockout of miR-137 exacerbated cerebral infarction and neuronal injury, while reducing learning and memory abilities in MCAO mice.

### Knockout of miR-137 increases oxidative stress and inflammation in the brain tissues of MCAO mice

**Figure 10:**
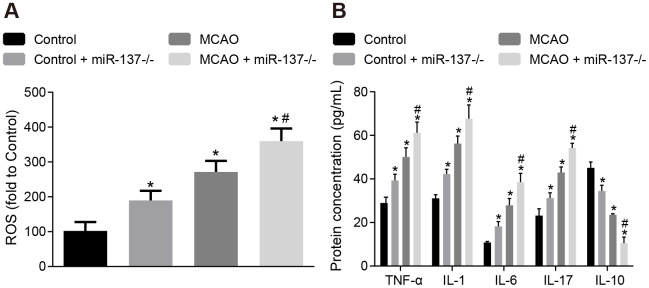
**Downregulation of miR-137 increases oxidative stress and inflammatory response in brain tissues from MCAO mice.** (**A**) ROS content in brain tissues from miR-137^-/-^ mice after staining with a DCFH-DA fluorescently-labeled probe; (**B**) TNF-α, IL-1, IL-6, IL-17 and IL-10 expression in brain tissues from miR-137^-/-^ mice using ELISA. Data are expressed as mean ± standard deviation and compared by one-way ANOVA, followed by Tukey's post hoc test. * *p* < 0.05 *vs*. control group (sham-operated wild-type mice); # *p* < 0.05 *vs*. MCAO group (wild-type mouse models of MCAO). N = 15.

Afterwards, levels of ROS and inflammatory factors in brain tissues were detected. The concentration of ROS and the expression of TNF-α, IL-1, IL-6 and IL-17 were increased (Figure 10A, 10B), whereas IL-10 expression was decreased in brain tissues of wild-type mouse models of MCAO in comparison to sham-operated wild-type mice (*p* < 0.05). The results were similar in miR-137^-/-^ mice (*p* < 0.05). These data indicate that inhibition of miR-137 augmented oxidative stress and inflammation in MCAO mice.

### Knockout of miR-137 activates the MAPK signaling pathway by elevating *Src* resulting in increased cell apoptosis

**Figure 11:**
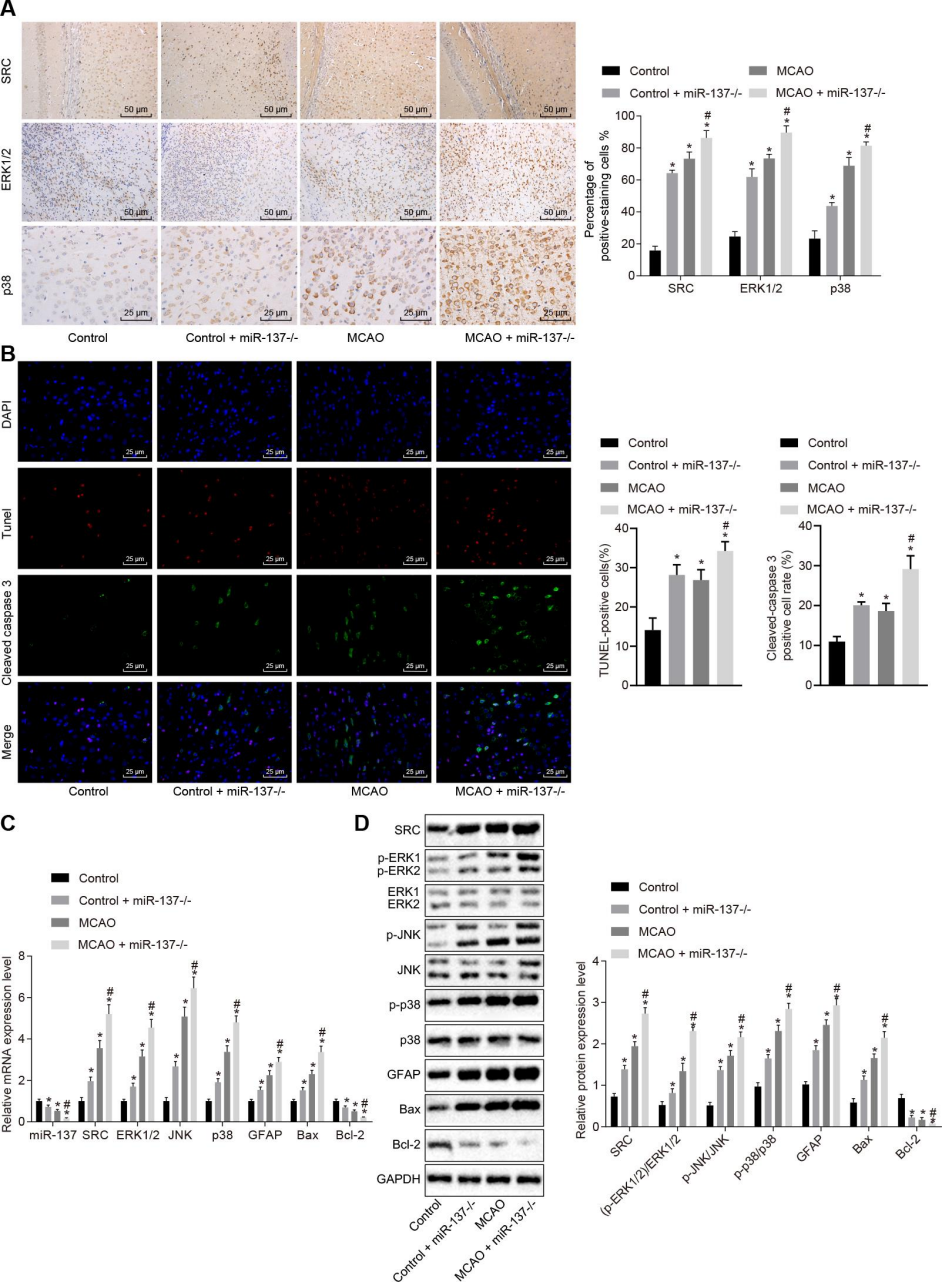
**Downregulated miR-137 activates the MAPK signaling pathway and increases cell apoptosis by increasing *Src* expression.** (**A**) ERK1/2, p38 and Src in the brain tissues of miR-137^-/-^ mice by immunohistochemistry (× 200); (**B**) immunofluorescence images of cleaved caspase 3, TUNEL staining, and DAPI staining in brain tissues from miR-137^-/-^ mice (× 400); (**C**) mRNA levels of *Erk2*, *Jnk*, *p38*, *Gfap*, *Bax* and *Bcl-2* in brain tissues from miR-137^-/-^ mice detected using RT-qPCR; (**D**) protein levels of GFAP, Bax and Bcl-2 along with the extent of ERK1/2, JNK and p38 phosphorylation in brain tissues from miR-137^-/-^ mice detected using Western blot analysis. Measurement data are expressed as mean ± standard deviation and compared using one-way ANOVA, followed by Tukey's post hoc test. * *p* < 0.05 *vs.* control group (sham-operated wild-type mice); # *p* < 0.05 *vs.* MCAO group (wild-type mouse models of MCAO). N = 15.

Immunohistochemistry was performed to identify the roles of miR-137 in the MAPK signaling pathway. The results showed an increase in positive staining for ERK1/2, p38 and Src in MCAO-operated miR-137^-/-^ mice (Figure 11A) (*p* < 0.05) compared with sham-operated wild-type mice. Analysis of cleaved caspase 3, TUNEL, and DAPI staining (Figure 11B) revealed that the number of Cleaved caspase 3 and TUNEL double positive cells increased in MCAO-operated miR-137^-/-^ mice in comparison to sham-operated wild-type mice (*p* < 0.05). RT-qPCR and Western blot analysis (Figure 11C, 11D) showed an increase in Src, ERK1/2, JNK, p38, GFAP and Bax expression as well as increased ERK1/2, JNK and p38 phosphorylation in the absence of miR-137^-/-^ while expression of miR-137 and Bcl-2 was reduced (*p* < 0.05). These results suggest that knockout of miR-137 activated the MAPK signaling pathway, upregulated *Gfap* and *Bax*, and downregulated *Bcl-2* by activating *Src*, and promoted cell apoptosis.

### miR-137 disrupts the MAPK signaling pathway by targeting *Src*

Subsequently, we attempted to verify that miR-137 inhibits the MAPK pathway by targeting *Src* expression *in vitro*. First, we designed three siRNAs to downregulate the *Src* gene, and infected astrocytes with lentiviruses, after which RT-qPCR was employed to detect the expression of *Src* in the astrocytes. The results showed that *Src* expression was reduced in cells upon treatment with si-*Src*-1, si-*Src*-2 and si-*Src*-3, among which si-*Src*-1 exhibited the lowest *Src* expression (Supplementary Figure 2). Thus, we chose the si-*Src*-1 sequence for subsequent experiments.

**Figure 12:**
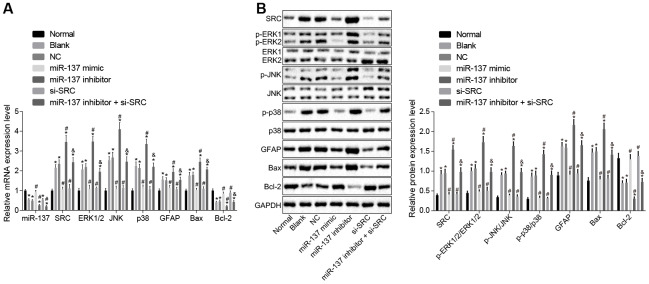
**miR-137 inhibits *Src* thus inactivating the MAPK signaling pathway in astrocytes.** (**A**) miR-137 expression and mRNA levels of *Src*, *Erk2*, *Jnk*, *p38*, *Gfap*, *Bax* and *Bcl-2* in astrocytes detected using RT-qPCR; (**B**) protein levels of Src, GFAP, Bax and Bcl-2 along with the extent of ERK1/2, JNK and p38 phosphorylation in astrocytes detected using Western blot analysis. Measurement data are expressed as mean ± standard deviation and compared using one-way ANOVA, followed by Tukey's post hoc test. * *p* < 0.05 *vs*. normal astrocytes; # *p* < 0.05 *vs*. OGD-induced astrocytes treated with negative controls; & *p* < 0.05 *vs*. astrocytes treated with miR-137 inhibitor. The experiment was repeated 3 times independently.

Next, RT-qPCR and Western blot analysis were performed to determine the expression of the MAPK signaling pathway-related genes in cell (Figure 12A, 12B). In comparison to normal astrocytes, astrocytes exposed to oxygen glucose deprivation (OGD) exhibited decreased levels of miR-137 and Bcl-2, but increased levels of Src, ERK2, JNK, p38, GFAP and Bax along with extent of ERK1/2, JNK and p38 phosphorylation (*p* < 0.05). Treatment with miR-137 mimic or si-*Src* eliminated the effects of OGD treatment on astrocytes (*p* < 0.05). These findings showed that the MAPK signaling pathway and *Src* were activated after OGD treatment and that miR-137 inhibited the *Src* and MAPK signaling pathways sequentially.

### Up-regulation of miR-137 or inhibition of *Src* inhibits inflammatory cytokine secretion and oxidative stress in astrocytes

**Figure 13:**
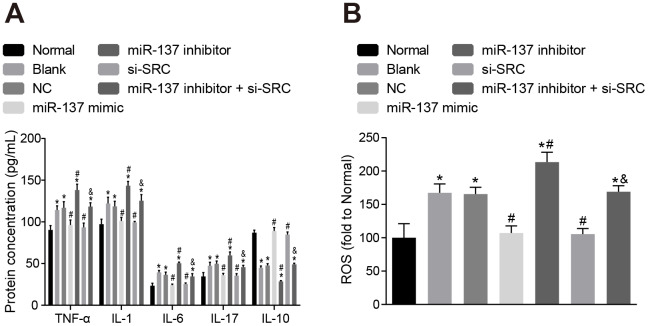
**Up-regulation of miR-137 or inhibition of *Src* inhibits the secretion of inflammatory cytokines and the degree of oxidative stress in astrocytes.** (**A**) The expression of inflammatory markers in astrocytes using ELISA; (**B**) quantitation of ROS in astrocytes for each treatment group using the DCFH-DA fluorescent probe. Measurement data were expressed as mean ± standard deviation and compared using one-way ANOVA, followed by Tukey's post hoc test. * *p* < 0.05 *vs*. normal astrocytes; # *p* < 0.05 *vs*. OGD-induced astrocytes treated with negative controls; & *p* < 0.05 *vs*. astrocytes treated with miR-137 inhibitor. The experiment was repeated 3 times independently.

Subsequently, we measured the expression of inflammatory factors and ROS and found that the expression of TNF-α, IL-1, IL-6, IL-17 and ROS was elevated (Figure 13A, 13B), while the expression of IL-10 was reduced in astrocytes exposed to OGD (*p* < 0.05). On the other hand, astrocytes treated with miR-137 mimic or si-*Src* showed opposite trends when compared with normal astrocytes (*p* < 0.05). These findings revealed that OGD treatment increased oxidative stress in cells and inflammation, while up-regulation of miR-137 or inhibition of *Src* reversed these effects in astrocytes.

### Up-regulation of miR-137 or inhibition of *Src* enhances viability and inhibits apoptosis of neuron cells

**Figure 14:**
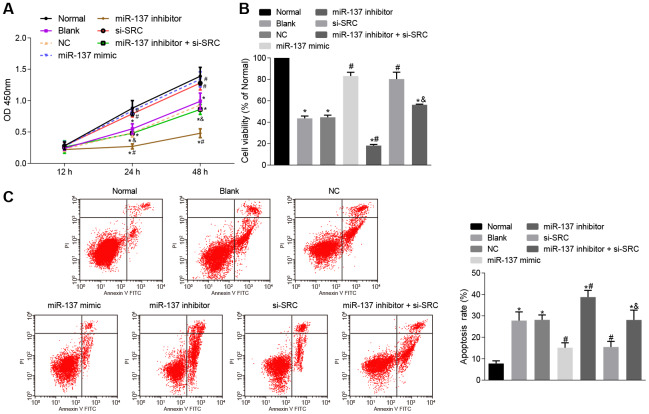
**Up-regulation of miR-137 or inhibition of *Src* suppresses apoptosis of neuron cells.** (**A**) neuronal cell viability after treatment with miR-137 mimic or si-*Src* measured using CCK-8; (**B**) neuronal cell activity after treatment with miR-137 mimic or si-*Src* measured using MTS assay; (**C**) apoptosis rate of neuron cells treated with miR-137 mimic or si-*Src* measured using Annexin V-FITC/PI double staining. Measurement data are expressed as mean ± standard deviation. Data among multiple groups are compared using one-way ANOVA, followed by Tukey's post hoc test and those among multiple groups at different time points were compared using two-way ANOVA, followed by Bonferroni's post hoc test. * *p* < 0.05 *vs*. normal astrocytes; # *p* < 0.05 *vs*. OGD-induced astrocytes treated with negative controls; & *p* < 0.05 *vs*. astrocytes treated with miR-137 inhibitor. The experiment was repeated 3 times independently.

Finally, cell counting kit-8 (CCK-8) assay (Figure 14A), 3-(4,5-dimethylthiazol-2-yl)-5(3-carboxymethonyphenol)-2-(4-sulfophenyl)-2H-tetrazolium (MTS) assay (Figure 14B) and Annexin V-fluorescein isothiocyanate (FITC)/propidium iodide (PI) double staining (Figure 14C) were used to assess the roles of miR-137 and *Src* in neuron cells. Astrocytes exposed to OGD had decreased viability and neuronal activity in comparison with normal astrocytes, while astrocytes treated with miR-137 mimic or si-*Src* showed opposite trends (*p* < 0.05). These findings suggest that overexpression of miR-137 or inhibition of *Src* could potentially enhance cell viability and reduce apoptosis in neuronal cells.

## DISCUSSION

Stroke impacts not only the nervous system, but also creates systemic problems that can lead to atherosclerosis, inflammation or infection, which may eventually result in infarction in other organs and manifest as heart attack [19]. In the current study, we provided evidence that up-regulated miR-137 down-regulates the *Src*/MAPK signaling pathway and is associated with a reduction in inflammation and oxidative stress leading to, reduced neuronal injury and cognitive impairment following ischemic stroke in a mouse model.

Animal and *in vitro* studies have shown that miRNAs participate in the pathogenesis of stroke by regulating oxidative stress, apoptosis, inflammation, and endothelial function [20]. Our study revealed that miR-137 knockout led to elevated oxidative stress and inflammation in MCAO mice, thereby promoting neuronal injury and cognitive impairment and reducing learning and memory abilities in mice. The expression of miR-137 is down-regulated in the MCAO-injured brain and in OGD-stimulated primary brain neurons and its upregulation can alleviate effects of growth arrest-specific 5 (GAS5) on the progression of ischemic stroke [21]. Inflammation levels and oxidative stress are found to be significantly higher after spinal cord injury and were reduced after transfection of miR-137, thus attenuating the impact of spinal cord injury [22]. In addition, overexpression of miR-137 enhances viability and suppresses apoptosis of neuronal cells, decreases cleaved caspase 3 levels and increases Bcl-2 protein levels in a cell model of Alzheimer's disease, highlighting its neuronal protective effects [23], which was in accordance with our findings.

MAPK is an essential signaling pathway involved in the regulation of cell apoptosis, proliferation, differentiation, senescence and migration [24]. The present study found that inhibition of *p38* or *Erk2* alleviated cerebral infarction, neuron damage, cognitive impairment, and inhibited oxidative stress, inflammatory response and cell apoptosis in MCAO mice. Similarly, down-regulation of the MAPK signaling pathway caused by oxysophocarpine, an alkaloid, reduced OGD-induced hippocampal neuron injury by attenuating the expression of inflammatory factors [25]. Also, down-regulation of the MAPK signaling pathway is thought to decrease lipopolysaccharide (LPS)-induced pro-inflammatory responses, thus reducing microglia-mediated neuronal damage [26]. Oxidative stress, which can promote the death of neuronal cells, is associated with various chronic neurodegenerative diseases and the toxicity induced by oxidative stress is associated with the activation of intracellular signaling pathways, such as of JNK and p38 [27]. Furthermore, a current study demonstrated that cerebral ischemia/reperfusion injury could be alleviated via down-regulation of the p38 MAPK-ATF2 signaling pathway, ultimately contributing to the suppression of neuronal apoptosis [28].

Emerging evidence demonstrates that miRNAs play an important role in regulation of disease, cell growth, invasion and metastasis by inhibiting the expression of their targets [29]. We identified *Src* as a target gene of miR-137 and confirmed its downregulation by miR-137 using *in silico* analysis. Likewise, miR-137 has been reported to target *Src* gene and inhibit its expression in colon cancer cells, slowing cancer progression [11]. Inhibiting *Src* activity also slows the progression of transient global cerebral ischemia [30]. Consistent with our results, knockout of *Src* alleviated cerebral infarction and neuronal injury in MCAO mice. Moreover, *Src* induced the activation of the p38-MAPK/JNK signaling pathway in CD34+ cells [31]. Thyroid cancer cells transfected with miR-137 mimics showed a reduction in p-ERK and p-AKT [32], suggesting a negative correlation between miR-137 and the MAPK signaling pathway. These results support our conclusion that miR-137 suppressed ischemic stroke progression by targeting *Src* gene thus blocking the MAPK signaling pathway.

**Figure 15:**
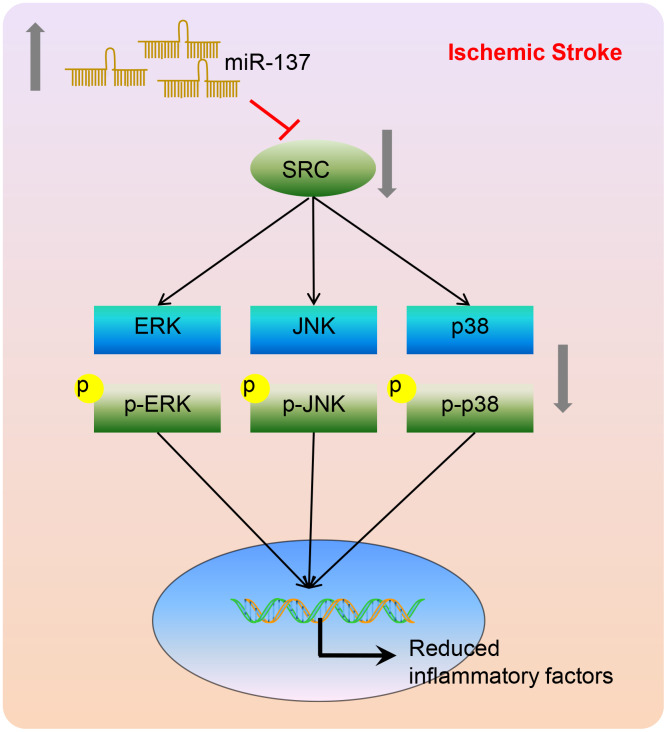
**A model depicting the mechanism by which miR-137, Src, and MAPK signaling interact to regulate the response to ischemic stroke.** miR-137 targets the *Src* gene resulting in the inactivation of the MAPK signaling pathway and reducing the inflammatory response and oxidative stress, thus alleviating ischemic stroke.

To conclude, miR-137 targeted the *Src* gene inactivating the MAPK signaling pathway. This leads to a reduction in inflammation and oxidative stress as well as a reduction in neuronal injury and cognitive impairment after ischemic stroke (Figure 15). This suggests that miR-137 manipulation may represent a potential therapeutic pathway for the treatment of ischemic stroke. A greater understanding of miR-137 regulated pathways during the onset of ischemic stroke is necessary to reveal innovative targets for drug discovery, biomarker development, and disorder modification. Nonetheless, whether *Src* is the only regulator of MAPK signaling in the ischemic stroke deserves further investigation so as to explain the specific underlying mechanism by which the miR-137/*Src*/MAPK network influences ischemic stroke.

## MATERIALS AND METHODS

### Ethics statements

This study was approved by the Experimental Animal Ethics Committee of The First Hospital of Jilin University. Animal experiments were performed in strict accordance to the Guide for the Care and Use of Laboratory animals published by the US National Institutes of Health.

### Microarray-based gene expression profiling

Microarray data associated with ischemic stroke (GSE9391) were downloaded from the Gene Expression Omnibus (GEO) Database (http://www.ncbi.nlm.nih.gov/geo). Standard pretreatment of the microarray data was conducted using the "limma" package in R language. The minimum criteria for identifying differentially expressed genes (DEGs) were a *p value <* 0.05 and |LogFoldChange| > 2. The expression of the DEGs was used to generate a heat map. Genes of interest in the String database were then analyzed for their ability to interact (interaction score > 0.4), followed by analysis of the Kyoto Encyclopedia of Genes and Genomes (KEGG) for gene enrichment. The Cytoscape 3.6.0 software was used to visualize the protein-protein interaction (PPI) network of the DEGs. miRNAs that potentially regulate DEGs were analyzed using the DIANA (http://diana.imis.athena-innovation.gr/DianaTools/index.php?r=microT_CDS/index), TargetScan (http://www.targetscan.org/vert_71/) and microRNA databases (http://34.236.212.39/microrna/getGeneForm.do). Finally, miRNAs identified using the prediction software were compared using software that generates custom Venn diagrams (http://bioinformatics.psb.ugent.be/webtools/Venn/).

### MCAO model establishment in mice

miR-137^-/-^ C57BL/6J male mice, *Src*^-/-^ C57BL/6J male mice, and wild-type C57BL/6J male mice (weighing 18-22 g, aged 8-10 weeks) were purchased from The Jackson Laboratory (Bar Harbor, Maine, USA) and housed in a non-specific-pathogen free (SPF) laminar shelf at 24-26°C with a constant humidity of 45-55%. Both the food and drinking water were sterilized before use.

A mouse model of MCAO was established by the thread occlusion method [33]. In brief, the mice were placed under anesthesia by intraperitoneal injection of 2% pentobarbital sodium (40 mg/kg), and then fixed. The left common carotid artery, external carotid artery and internal carotid artery were exposed through a 1 cm incision in the neck. The proximal part of the common carotid artery and distal end of external carotid artery were ligated. A loose knot was tied between the two nodes in the common carotid artery, and the thread was inserted into the internal carotid artery from the external carotid artery and then reached the middle cerebral artery (about 12 mm). One hour after obstruction of the blood vessels, the thread was taken out and the skin was sutured. The mice in the sham-operated group were operated on but no knot was made.

Lentiviral constructs were then injected into the lateral ventricle of mice. In short, *p38* siRNA, *Erk2* siRNA and control lentiviral constructs were purchased from Shanghai Genechem Co., Ltd. (Shanghai, China). The mice were anesthetized by injection with 2% pentobarbital sodium (40 mg/kg) in the head, and then injected with 10 μL siRNA lentivirus (a titer of 2 × 10^8^ U/mL) in the skull. The follow-up experiment was carried out 7 days later.

### Animal grouping

The animal experiment was divided into three parts. (1) The first part was to identify effects of p38 and ERK1/2 signaling pathway on ischemic injury in MCAO mice. (2) The second part was to investigate whether *Src* knockout affected ischemic injury in MCAO mice by inhibiting the MAPK signaling pathway. (3) Third, we determined whether miR-137 targeted the *Src* gene during ischemic injury in MCAO mice. Following the MCAO procedure, 1-4 mice in each group died, while mice that didn’t receive MCAO survived normally. The total number of mice was 190, of which 17 died and 8 mice were used for alternatives, with a total survival rate of 91.05%. The specific grouping was as follows. A total of 75 wild-type mice were divided into control mice (receiving blank lentivirus and sham operation) and MCAO mice (receiving blank lentivirus and MCAO operation), which were further treated with si-*p38*, si-*Erk2* or in combination. In addition, 90 *Src*^-/-^ mice were divided into control (*Src*^-/-^ mice receiving sham operation) and MCAO + *Src*^-/-^ mice (*Src*^-/-^ mice receiving MCAO operation). Also, 90 miR-137^-/-^ mice were divided into control (miR-137^-/-^ mice receiving sham operation) and MCAO + miR-137^-/-^ mice (miR-137^-/-^ mice receiving MCAO operation).

### Nerve function scoring

Nerve function of the mice was analyzed using Longa score at 48 h post operation, and score of neurological function was recorded [34].

### Foot-fault test

The foot-fault test was performed at 48 h post operation. The test indexes included number of right foot fault and left foot fault. During the first 3 days of the experiment, the mice were trained to climb across an elevated network from one end to the other. The foot-fault test was performed on the first and third days after the training and the fault step rate was calculated [35].

### Step-down test

The test box was 60 cm × 50 cm × 60 cm, and the bottom was stainless steel strip with an electric device. Food was placed in front of the plastic platform. The box was connected to power supplies (40 V for 2 s) when the mice ate the food. The duration of time from when the mice jumped onto the platform, where the food was provided, until they jumped down, the “latent period”, during which the mice received electric shock, was recorded. The number of times the mouse received electric shock during a 3 min period was recorded as the fault number. The test was performed after training for several min in a quiet environment [36].

### Nissl staining

Seven days after operation, the mice were euthanized by anesthesia. After cardiac perfusion, the brains of mice were fixed in 10% formaldehyde, embedded in paraffin and sliced at a thickness of 4 μm. The samples were dehydrated with 100% absolute ethyl alcohol for 10 min and 95% ethanol for 2 min. Next, the slices were treated with Nissl staining for 10 min at 50°C, separated using 95% ethanol for 20 s, dehydrated using 100% absolute ethyl alcohol for 5 min, and mounted using neutral gel. Histopathological changes were then observed and images were obtained under a common microscope (Nikon Corporation, Tokyo, Japan).

### TTC staining

Brain tissues were frozen at -20°C for 20 min and then successive coronal slices (1.5 mm thick) were made. Then the slices were incubated with 0.5% TTC solution at 37°C in the dark. The brain slices were scanned. The images were optimized and processed, and the infarct size was then analyzed using the Image-pro plus software (Media Cybernetics, Silver Spring, MD, USA).

### Measurement of ROS accumulation

The brain tissues of the mice were collected seven days after MCAO operation and the cerebral cortex of the ischemic side was separated on ice, added with pre-cooled homogenate buffer at a ratio of 1: 10, ground into tissue homogenate on ice, and then centrifuged to obtain the supernatant. Then, 10 μmol/L 2’,7’-Dichlorodihydrofluorescein diacetate (DCFH-DA) fluorescent probe was mixed with the tissue homogenate or astrocyte culture medium in a 37°C cell incubator for 20 min, and then rinsed with phosphate buffer saline (PBS) three times to remove the probe. The fluorescence level of DCFH in tissues was detected using an automatic microplate reader. The fluorescence level was measured at an excitation wavelength of 488 nm and an emission wavelength of 525 nm.

### Enzyme-linked immunosorbent assay (ELISA)

The expression of inflammatory factors in the supernatant derived from mice brain homogenate or astrocytes was detected using TNF-α, IL-1, IL-6, IL-10 and IL-17 ELISA kits according to the manufacturer’s instructions (Wuhan Beinglay Biotechnology Co., Ltd., Hubei, China).

### Immunohistochemistry

Seven days after operation, paraffin-embedded cerebral cortex tissues in the area surrounding the infarction were sliced, fixed, cleared, and mounted. Next, the tissue slices were incubated with anti-mouse ERK1/2 monoclonal antibody (1: 100, ab54230), anti-mouse p38 monoclonal antibody (1: 200, ab31828), or anti-rabbit *Src* monoclonal antibody (1: 500, ab109381 (Abcam, Cambridge, UK) at 4°C. Next, the slices were incubated with secondary antibody working solution of goat anti-rabbit or anti-mouse (ZSGB-BIO, Beijing, China) at 37°C for 1 h, and then developed using 3, 3'-diaminobenzidine (DAB) (ZSGB-BIO, Beijing, China) for 3-5 min. True color multi-functional cell image analysis system (Media Cybernetics, Silver Spring, MD, USA) was used to record images. Four slices were taken from each specimen, and 6 fields were randomly selected. The percentage of positive cells was counted under a light microscope and the average value was taken to reflect the positive level of ERK1/2, Src or p38, respectively.

### Immunofluorescence assay

Cerebral cortex tissues on the infarction side were sliced, rinsed, fixed, cleared, and then incubated with mouse anti-cleaved caspase 3 (ab49822; 1: 1000) at 4°C overnight. The slices were then incubated with TRITC-labeled rabbit anti-goat secondary antibodies (1: 1000, ab53554) at 37°C for 1 h in dark, incubated with DAPI (Thermo Fisher Scientific, San Jose, California, USA) at 37°C for 1 h, and then mounted by addition of anti-quenching sealing agent. Images were acquired with a laser-scanning confocal microscope in 5 randomly selected fields from each slice. All above antibodies were purchased from Abcam (Cambridge, UK). TUNEL staining was performed according to the manufacturer’s instructions included with the TUNEL staining kit (Roche Ltd., Basel, Switzerland). Images were acquired with a fluorescence microscope in 5 randomly selected fields on each slide. TUNEL-positive cells are shown in red and DAPI staining in blue indicating all cells. Image J software was employed to count the number of positive cells under the same magnification and the percentage of TUNEL-positive cells among all cells was calculated, followed by statistical analysis.

### RNA isolation and quantitation

Total RNA was collected from mouse brain tissues or astrocytes using an RNA Extraction Kit (10296010, Invitrogen, Co., Ltd., Shanghai, China). RNA was then reverse transcribed into complementary DNA and antisense miRNA according to the instructions in the PrimeScript RT kit (RR014A, Takara Biotechnology Ltd., Beijing, China). Primers (Table 1) were designed and synthesized by Takara Biotechnology Ltd. (Beijing, China). RT-qPCR was carried out using the PCR reagent kit (KR011A1, Tiangen Biotechnology Co., Ltd., Beijing, China). *U6* served as the internal reference for miR-137 and glyceraldehyde-3-phosphate dehydrogenase (*Gapdh*) served as the internal reference for the remaining genes. The fold changes were calculated based on the relative quantification 2^-ΔΔCt^ method [37].

**Table 1:** Primer sequences for RT-qPCR.

**Genes**	**Primer sequences (5’-3’)**	
	**Forward**	**Reverse**
miR-137	TTATTGCTTAAGAATACGCG	TCGTATCCAGTGCAGGGTC
*Src*	CTGAGCAGATGAATGATCCA	GGACGTCAGCAAACACCTGA
*Erk2*	ACAGGACCTCATGGAGACGG	GATCTGCAACACGGGCAAGG
*Jnk*	ATTGAACAGCTCGGAACACC	GAGTCAGCTGGGAAAAGCAC
*p38*	TCACGCAAAAGGACCTACC	ATTCCTCCAGTGACCTTGCG
*Gfap*	CGGAGACGCATCACCTCTG	TGGAGGAGTCATTCGAGACAA
*Bax*	CTGAGCTGACCTTGGAGC	GACTCCAGCCACAAAGATG
*Bcl-2*	GACAGAAGATCATGCCGTCC	GGTACCAATGGCACTTCAAG
*U6*	GCTCGCTTCGGCAGCACAT	AAAATATGGAACGCTTCACG
*Gapdh*	ACCACAGTCCATGCCATCAC	TCCACCACCCTGTTGCTGTA

### Western blot analysis

The ischemic brain tissues (obtained 7 days after operation) or astrocytes were collected and lysed in radioimmunoprecipitation assay (RIPA) buffer (PS0013, Reagan Biotechnology Co., Ltd., Beijing, China). The proteins were then separated by sodium dodecyl sulfate-polyacrylamide gel electrophoresis (SDS-PAGE) and transferred to a nitrocellulose membrane. Subsequently, the membrane was incubated overnight at 4°C with diluted primary rabbit anti-mouse antibodies against *Src* (1: 1000, ab47405), ERK1/2 (1: 1000, ab184699), p-ERK1/2 (1:1000, ab201015), JNK (1: 1000, ab179461), p-JNK (1: 1000, ab124956), p38 (1: 1000, ab170099), p-p38 (1: 1000, ab195049), GFAP (1: 10000, ab68428), Bax (1: 1000, ab32503), Bcl-2 (1: 1000, ab59348) or GAPDH (1: 5000, ab22555) (Abcam, Cambridge, UK). After being rinsed with PBS containing 0.1% Tween-20 (PBST) (10 min, 3 times), the membrane was incubated with horseradish peroxidase-labeled secondary goat anti-rabbit (1: 10000, DF109489, Yaoyun Biotechnology Co., Ltd., Shanghai, China) for 1 h at room temperature. The immunocomplexes on the membrane were visualized using enhanced chemiluminescence (ECL) reagent (36208ES60, Amersham Life Sciences Inc., Arlington Heights, Illinois, USA). ImageJ software was used for semi-quantitative analysis of Western blot results. The relative level of the protein was calculated using the ratio between the target band and the internal reference band. Experiments were repeated three times.

### Dual-luciferase reporter assay

The *Src* 3’UTR gene fragment was artificially synthesized, and then introduced into pMIR-reporter plasmid (Promega, Madison, WI, USA) within the endonuclease site SpeI and Hind III. After restriction enzyme cutting, wild-type sequence and a mutant target fragment of *Src* were inserted into the pMIR-reporter reporter plasmid using T4 DNA ligase. Wild-type *Src* luciferase reporter plasmid and mutant luciferase reporter plasmids were co-transfected with miR-137 into HEK-293T cells (Shanghai Beinuo Biotechnology Co., Ltd., Shanghai, China) respectively for 48 h. Next, the cells were collected. Luciferase activity in cell extracts was analyzed by Dual-Luciferase Reporter Assay System (Promega Corp., Madison, Wisconsin, USA).

### *In situ* hybridization

The cerebral cortex on the ischemic side of the control group and the MCAO group was mounted on a slide, incubated at 60°C for 1 h, dewaxed and hydrated by xylene I and xylene II for 30 min respectively, and then dehydrated with gradient alcohol. An *in situ* hybridization Kit (Wuhan Boster Biological Technology Ltd., Hubei, China) was used to detect miR-137 expression in mouse brain tissues. The probe was synthesized by Shanghai Invitrogen, Co., Ltd. (Shanghai, China). *In situ* hybridization was performed according to the standard protocol included with the kit. Images were analyzed using multi-functional cell image analysis management system (Media Cybernetics, Silver Spring, MD, USA). Four slices were taken from each specimen, and 3 fields were randomly selected from each slice. The percentage of positive cells was counted under a light microscope and the average value was calculated to reflect the positive level of miR-137.

### *In vitro* isolation and subculture of primary astrocytes and cerebral cortical neuron cells in mice

C57BL/6J mice no more than 3 days old were purchased from Shanghai SLAC Laboratory Animal Co., Ltd. (Shanghai, China) and euthanized by decapitation under aseptic conditions. After removal of the pia mater and blood vessels, the cerebral cortex was obtained, and treated with 0.125% trypsin at 37°C for 15 min. Trypsinization was terminated using complete medium. The mixture was filtered, centrifuged at 1000 rpm to obtain the precipitate and then complete medium was added to make a single cell suspension. Subsequently, the cells were inoculated in 75 mm^2^ culture bottles coated with poly-L lysine (5 × 10^5^ cells/cm^2^), and then cultured in a 37°C incubator with 5% CO_2_. The culture medium for astrocytes was changed for the first time after 24 h, and then every 2 days, thereafter. When cells reached 80-90% confluence, they were subcultured at a density of 1 × 10^5^ cells/mL, and then cultured in high-glucose Dulbecco's modified Eagle's medium (DMEM) supplemented with 10% fetal bovine serum. Neuronal cell culture medium was replaced by Neurobasal medium supplemented with 2% B27 (GIBCO BRL, Gaithersburg, MD, USA). After 48 h, 5 μg/mL cytosine arabinoside (Sigma, USA) was added to inhibit the growth of astrocytes. After 48 h of incubation, the medium was changed, and then half of the medium was changed every day for 6-7 days for subsequent experiments.

### Identification of astrocytes

The cells were fixed with 4% paraformaldehyde for 15 min, treated with 1% Triton X-100 for 15 min to permeabilize the cells, and then incubated with 3% H_2_O_2_ in deionized water for 10 min to block endogenous peroxidase activity. After being blocked with 10% goat serum for 30 min, the cells were incubated with diluted goat anti-GFAP (1: 1000, ab4674) at 4°C overnight, and incubated with TRITC-labeled secondary goat anti-rabbit (1: 1000, ab53554) at 37°C for 1 h in the dark. The cells were observed under a fluorescence microscope. All above antibodies were purchased from Abcam (Cambridge, UK).

### Establishment of OGD-induced astrocyte model

OGD model was prepared using well-grown astrocytes. The medium was replaced with sugar free medium, and put into a 37°C hypoxic incubator containing 5% CO_2_, 2% O_2_ and 93% N_2_ for 16 h. Subsequently, the medium was replaced with high glucose medium with 10% fetal bovine serum, and then incubated in a saturated normoxic incubator at 37°C with 5% CO_2_.

### Astrocyte treatment

miR-137 mimic, miR-137 inhibitor and negative controls were purchased from Shanghai Reborn Biological Engineering Co., Ltd. (Shanghai, China). Lentivirus containing *Src* siRNA was purchased from Shanghai Genechem Co., Ltd. (Shanghai, China). Cells were incubated in a 5% CO_2_ incubator at 37°C, and then transfected with one of the above miRNAs, respectively after cells reached 80% confluence. Lipofectamine^TM^ 2000 and miR-137 mimic or miR-137 inhibitor were prepared according to the Lipofectamine 2000 instructions (Invitrogen, Carlsbad, USA). After transfection for 48 h, the cells were collected and used in subsequent experiments. After even trituration, the 6-well plate was placed in a 5% CO_2_ incubator at 37°C for 48 h.

The cells were treated with si-*p38*-1 (cells infected with *p38* siRNA-1 lentivirus), si-*p38*-2 (cells infected with *p38* siRNA-2 lentivirus), si-*p38*-3 (cells infected with *p38* siRNA-3 lentivirus), si-*Erk2*-1 (cells infected with *Erk2* siRNA-1 lentivirus), si-*Erk2*-2 (cells infected with *Erk2* siRNA-2 lentivirus), si-*Erk2*-3 (cells infected with *Erk2* siRNA-3 lentivirus), miR-137 mimic (cells transfected with miR-137 mimic for 48 h and treated with OGD for 16 h), miR-137 inhibitor (cells transfected with miR-137 inhibitor and treated with OGD for 16 h), si-Src (cells infected with Src siRNA lentivirus and treated with OGD for 16 h) and miR-137 inhibitor + si-Src (cells transfected with miR-137 inhibitor and infected with Src siRNA lentivirus and treated with OGD for 16 h) as well as their corresponding controls.

### Co-culture of astrocytes and neuron cells

Astrocytes were cultured in 6-well plates with 4 paraffin columns (2 mm height) at the bottom of each well to support the slide. Neurons were cultured on glass slides treated with poly-L-lysine for 7 days, and then co-cultured with astrocytes. Astrocytes and neurons were collected for subsequent experiments after 48 h of culture.

### CCK-8 assay

Neuronal cells were seeded in a 96-well plate at a density of 5 × 10^4^ cells/mL and then incubated at 37°C with 5% CO_2_. Three wells of each group were randomly selected for addition of 10 μL of CCK-8 (Dojindo Laboratories, Kumamoto, Japan) at 37°C for 4 h at 12 h, 24 h or 48 h. Optical density (OD) of each well was measured at 450 nm using an enzyme-linked immunometric meter (elx800, Bio-Tek, Vermont, USA). Experiments were repeated three times.

### MTS assay

Neuronal cells were seeded in a 96-well plate at a density of 5 × 10^4^ cells/mL and treated with 20 μL of Cell Titer 96 AQueous One Solution Reagent and then incubated in a 37°C saturated humidity incubator for 1 h. OD of each well was measured at 490 nm using a microplate reader. Cell survival rate was then calculated.

### Annexin V-FITC/PI double staining

Neuronal cells were cultured in a 5% CO_2_ incubator at 37°C for 48 h. The cells were collected and resuspended in 200 μL binding buffer after centrifugation. The cells were then mixed with 10 μL Annexin V-FITC (ab14085, Abcam, Cambridge, UK) and 5 μL propidium iodine (PI) for 15 min avoiding exposure to light, and then 300 μL of binding buffer was added. Apoptotic cells were detected using a flow cytometry (Becton, Dickinson and Company, Franklin Lakes, NJ, USA).

### Statistical analysis

Statistical analyses were conducted using the SPSS 21.0 statistical software (International Business Machines Corporation., Armonk, NY, USA). All data were expressed as mean ± standard deviation. Measurement data between two groups were compared by independent sample *t*-test, while among multiple groups were compared by one-way analysis of variance (ANOVA), followed by Tukey's post hoc tests with corrections for multiple comparisons. Two-way ANOVA with Bonferroni post hoc test was applied for the comparison of data at different time points. Pearson's correlation coefficient was used for correlation analysis. *p* < 0.05 was indicative of statistical significance.

## Supplementary Material

Supplementary Figures
