# Correction for: miR-137 prevents inflammatory response, oxidative stress, neuronal injury and cognitive impairment via blockade of *Src*-mediated MAPK signaling pathway in ischemic stroke

**DOI:** 10.18632/aging.202509

**Published:** 2021-01-15

**Authors:** Runhui Tian, Bo Wu, Cong Fu, Kaimin Guo

**Affiliations:** 1 Department of Psychology, The First Hospital of Jilin University, Changchun, 130021, P.R. China; 2 Department of Psychology, The Sixth People's Hospital of Changchun, Changchun, 130000, P.R. China; 3 Department of Andrology, The First Hospital of Jilin University, Changchun, 130021, P.R. China

**Keywords:** Correction

**This article has been corrected:** The authors made the mistake in Funding information. The correct Funding is provided below. This correction does not change the content of the publication.

Original article: Aging. 2020; 12:10873–10895. . https://doi.org/10.18632/aging.103301

