# Supplementary material for: miR-137 prevents inflammatory response, oxidative stress, neuronal injury and cognitive impairment via blockade of Src-mediated MAPK signaling pathway in ischemic stroke
Source: Aging (Albany NY). 2020 Jun 4;12(11):10873–95. doi: 10.18632/aging.103301 (PMC7346022; doi:10.18632/aging.103301)
Supplement: Supplementary Figures [file aging-12-103301-s001..pdf]

SUPPLEMENTARY FIGURES

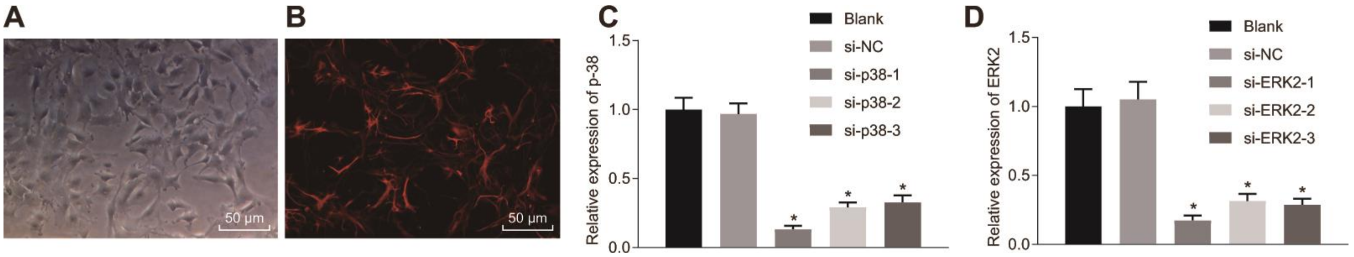

Supplementary Figure 1. Efficiency of *p38* or *Erk2* knockdown in astrocytes. (**A**) the morphological characteristics of astrocytes observed under a microscope (× 200); (**B**) immunofluorescence analysis of astrocytes (× 200); (**C**) expression of *p38* in astrocytes detected by RT-q-PCR; (**D**) expression of *Erk2* in astrocytes detected by RT-q-PCR. Data are expressed as mean ± standard deviation and compared using one-way ANOVA, followed by Tukey's post hoc test. * $p < 0.05$ *vs*. cells treated with si-NC. The experiment was repeated 3 times independently.

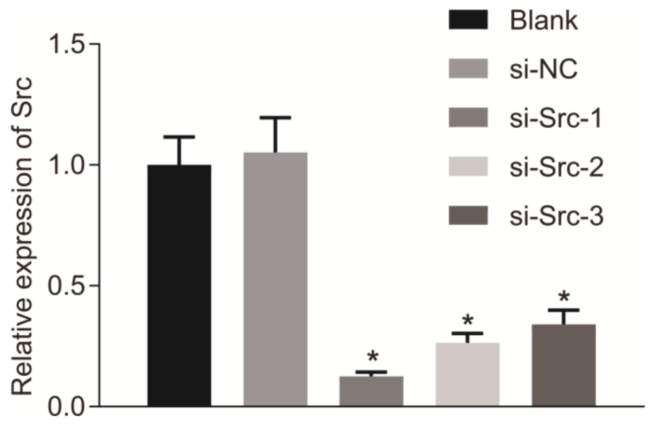

Supplementary Figure 2. Efficiency of *Src* knockdown in astrocytes. Data are expressed as mean ± standard deviation and compared using one-way ANOVA, followed by Tukey's post hoc test. * $p < 0.05$ *vs*. cells treated with si-NC. The experiment was repeated 3 times independently.
